# Remote Therapeutic Monitoring Use Among Commercially Insured and Medicare Advantage Patients

**DOI:** 10.2196/103600

**Published:** 2026-09-21

**Authors:** Joseph H Joo, Jessica I Billig, Nadia Lieu, Lingmei Zhou, Joshua M Liao

**Affiliations:** 1 Department of Internal Medicine, University of Texas Southwestern Medical Center, 5323 Harry Hines Blvd, Dallas, TX, 75390, United States, 1 (214) 648-311; 2 Program on Policy Evaluation and Learning, Dallas, TX, United States; 3 Department of Health Economics, Systems, and Policy, University of Texas Southwestern Medical Center, Dallas, TX, United States; 4 Department of Plastic Surgery, University of Texas Southwestern Medical Center, Dallas, TX, United States

**Keywords:** remote therapeutic monitoring, commercially insured, Medicare Advantage, patients with musculoskeletal conditions, RTM

## Abstract

This national study found that remote therapeutic monitoring use increased rapidly between 2022 and 2024 but remained low overall, varied by insurance type and clinician specialty, and was concentrated among patients with musculoskeletal conditions.

## Introduction

Remote therapeutic monitoring (RTM) enables the collection and monitoring of nonphysiological data, including functional status, treatment adherence, and patient-reported symptoms [1]. Through RTM, clinicians can gain insight into patients’ responsiveness to and engagement with prescribed therapies outside of traditional clinical settings. This information can help identify changes in clinical status that require early intervention, particularly for patients with musculoskeletal, respiratory, and oncologic conditions [2-5]. However, despite the potential for RTM to support proactive care and improve clinical outcomes, studies regarding its use remain limited. Therefore, the aim of our nationwide study was to describe RTM use nationwide among commercially insured and Medicare Advantage (MA) patients.

## Methods

### Overview

We used 2022‐2024 Merative MarketScan data, which contain longitudinal commercial and MA insurance claims in the United States, and identified RTM using Current Procedural Terminology codes (98975, 98976, 98977, 98980, 98981). Separately for commercial and MA claims, we assessed the total number of months of RTM use and median number of months of RTM use per patient. Patients were included if they had at least 1 month of RTM use and were enrolled in insurance for at least 11 out of 12 months in a calendar year.

We described the number and characteristics of patients using RTM with respect to age, sex, region, clinical complexity (ie, Charlson Comorbidity Index), and insurance plan type (eg, preferred provider organization, health maintenance organization) [6]. *International Classification of Diseases, Tenth Revision, Clinical Modification* codes were used to identify patient diagnoses for RTM use then grouped using Clinical Classification Software Refined categories (Multimedia Appendix 1) [7]. Lastly, RTM use was stratified by clinician and facility type as follows: primary care physicians (eg, internal medicine and family medicine providers), medical subspecialists (eg, cardiologists and pulmonologists), surgeons (eg, orthopedic and neurological surgeons), other physician specialists (eg, neurology and pain medicine specialists), advanced practice providers (eg, physician assistants and nurse practitioners), other clinicians (eg, therapists and nurses) and facilities (eg, rehabilitation or specialty care facilities) [8].

### Ethical Considerations

As this study used deidentified data and did not involve human subjects as defined by federal regulations, it was determined to be exempt by the institutional review board of the University of Texas Southwestern Medical Center.

## Results

Between 2022 and 2024, RTM was used by 16,116 patients (n=12,951 commercially insured and n=3165 MA patients; Table S1 in Multimedia Appendix 2). Use of RTM increased from 2089 months (n=1528 commercial and n=561 MA) in 2022 to 24,494 months (n=18,368 commercial and n=6126 MA) by 2024.

Commercially insured patients used RTM a total of 26,936 months, with a median of 1 (IQR 1‐2) month of RTM use per patient. With over 48 million patients enrolled during the study period, this equated to approximately 5.6 RTM months per 10,000 commercially insured patients. Most (12,280/12,951, 94.8%) used RTM during a single calendar year, while a minority (671/12,951, 5.2%) used RTM during multiple years. The majority of the 12,951 patients were female (8061/12,951, 62.2%), enrolled in a preferred provider organization (7855/12,877, 61%), and had low clinical complexity (12,801/12,951, 98.8%). Additionally, a plurality of patients were between the ages of 55 and 64 years (4116/12,951, 31.8%) and from the US South (6112/12,951, 47.2%).

Comparatively, MA patients used a total of 9551 RTM months, with a median of 2 (IQR 1‐3) months of RTM use per patient. With nearly 4 million patients enrolled between 2022 and 2024, there were about 24.3 RTM months per 10,000 MA patients. A large majority (2949/3165, 93.2%) used RTM during a single calendar year, and a minority (216/3165, 6.8%) used RTM across multiple years. Among the MA patients, the majority were 75 years or older (1654/3165, 52.3%), female (1982/3165, 62.6%), from the US North Central region (1775/3165, 56.1%), enrolled in a preferred provider organization (2619/2999, 87.3%), and had low clinical complexity (2768/3165, 87.5%).

Among patients with commercial insurance, 48.7% of RTM was delivered by other clinicians (eg, therapists, nurses), followed by other physician specialists (16.5%) and then advanced practice professionals (10.1%; Table 1). The predominant diagnosis category for RTM was diseases of the musculoskeletal system and connective tissue (56.3%). The next most common categories were diseases of the nervous system (17.2%), and factors influencing health status and contact with health services (5.5%).

**Table 1.** Percentage of remote therapeutic monitoring use across specialty–primary diagnosis combinations among commercial insurance beneficiaries.

	Other nonphysicians, %	Other physicians, %	Advanced practice providers, %	Surgeons, %	Primary care physicians, %	Facility-based, %	Medical subspecialists, %	Total, %
Diseases of the musculoskeletal system and connective tissue	34.62	7.47	4.11	5.04	1.60	2.40	1.05	56.28
Diseases of the nervous system	0.57	7.13	4.90	0.12	1.46	2.53	0.52	17.23
Factors influencing health status and contact with health services	1.32	0.64	0.53	0.67	0.47	0.06	1.83	5.52
Symptoms, signs, and abnormal clinical and laboratory findings	3.12	0.12	0.03	0.05	0.21	0.29	0.04	3.87
Diseases of the genitourinary system	3.71	0.02	0.01	0.01	0.06	0.04	0.01	3.86
Injury, poisoning, and certain other consequences of external causes	2.13	0.14	0.11	1.09	0.07	0.16	0.05	3.75
Endocrine, nutritional, and metabolic diseases	1.91	0.11	0.06	0.18	1.09	0.14	0.13	3.62
Diseases of the circulatory system	0.27	0.14	0.07	0.03	1.19	0.05	0.10	1.84
Mental, behavioral, and neurodevelopmental disorders	0.32	0.52	0.21	0.02	0.31	0.17	0.11	1.67
Diseases of the respiratory system	0.01	0.08	0.01	0.01	0.33	0.01	0.33	0.78
Other	0.74	0.08	0.05	0.11	0.35	0.09	0.17	1.59
Total	48.72	16.45	10.11	7.32	7.15	5.94	4.32	100.00

Among MA patients, RTM was most frequently delivered by primary care physicians (37.8%), other clinicians (23.9%), and other physician specialists (21.3%; Table 2). The primary diagnosis category associated with RTM was also diseases of the musculoskeletal system and connective tissue (45.0%), followed by diseases of the circulatory system (14.2%) and nervous system (10.9%).

**Table 2.** Percentage of remote therapeutic monitoring use across specialty–primary diagnosis combinations among Medicare Advantage beneficiaries.

	Primary care physicians, %	Other nonphysicians, %	Other physicians, %	Surgeons, %	Medical sub specialists, %	Facility-based, %	Advanced practice providers, %	Total, %
Diseases of the musculoskeletal system and connective tissue	8.29	19.35	8.07	4.22	1.22	2.54	1.32	45.02
Diseases of the circulatory system	9.17	0.22	4.18	0.03	0.41	0.22	0.00	14.23
Diseases of the nervous system	5.85	0.44	2.91	0.13	1.35	0.15	0.08	10.90
Endocrine, nutritional, and metabolic diseases	5.45	0.06	0.95	0.16	0.35	0.08	0.04	7.09
Symptoms, signs, and abnormal clinical and laboratory findings	1.01	2.22	1.16	0.00	0.07	0.54	0.00	5.01
Diseases of the respiratory system	2.32	0.05	1.43	0.02	0.29	0.14	0.02	4.27
Factors influencing health status and contact with health services	0.77	0.59	0.56	0.97	0.16	0.20	0.26	3.52
Diseases of the genitourinary system	0.62	0.24	0.74	0.00	0.01	0.04	0.00	1.65
Injury, poisoning, and certain other consequences of external causes	0.43	0.60	0.37	0.06	0.00	0.03	0.04	1.53
Mental, behavioral, and neurodevelopmental disorders	1.04	0.00	0.30	0.00	0.09	0.03	0.07	1.52
Other	2.86	0.11	0.61	0.05	1.54	0.04	0.05	5.26
Total	37.82	23.88	21.29	5.64	5.48	4.01	1.89	100.00

## Discussion

In this nationwide study, RTM use grew during the first 3 years after the billing codes were introduced, overall use remained low, and different use patterns were observed between commercially insured and MA patients. These early findings provide several observations to build on in future studies.

First, despite a smaller sample for MA versus commercial patients during the study period, RTM use was approximately 4 times higher among MA patients (24.3 versus 5.6 months per 10,000). Because MA patients are older with more comorbidities (12.5% for MA vs 1.2% for commercially insured patients with a Charlson Comorbidity Index of ≥2), it might be expected that RTM was used more among MA patients. Given that MA’s goal is to improve longitudinal outcomes through managed care, future work should assess whether more frequent RTM use among MA patients is associated with improved patient outcomes and lower spending. How such results combine with the findings of this and prior studies (eg, remote monitoring use for several months mostly among older female patients) remains within the purview of future work [9,10].

Second, RTM use among both commercial and MA patients was dominated by diseases of the musculoskeletal system and connective tissue, underscoring the role of therapeutic monitoring in supporting rehabilitation and functional monitoring. However, our results showed differences in those delivering RTM. Among commercial insurance patients, RTM was most frequently delivered by clinicians such as therapists and nurses, while among MA patients, RTM was more frequently delivered by primary care physicians. Future studies could evaluate the underlying incentives, approaches, and effectiveness of RTM when delivered through different clinician approaches.

Study limitations included the descriptive design and the inability to evaluate associations between RTM use and clinical outcomes. Although our study period included over 50 million patients, our analysis does not reflect RTM use among traditional Medicare patients. Nonetheless, we believe our study provides valuable observations of patterns in RTM use and, in turn, foundational insights for future research.

## Supplementary material

10.2196/103600 Multimedia Appendix 1 Clinical Classifications Software Refined categories for *International Classification of Diseases, Tenth Revision, Clinical Modification* diagnoses.

10.2196/103600 Multimedia Appendix 2 Characteristics of commercially insured and Medicare Advantage patients using remote therapeutic monitoring, as well as clinician specialties and facilities.

## Abbreviations

MA: Medicare Advantage
RTM: remote therapeutic monitoring
