# Supplementary material for: Remote Therapeutic Monitoring Use Among Commercially Insured and Medicare Advantage Patients
Source: JMIR Rehabil Assist Technol. 2026 Sep 21;13:e103600. doi: 10.2196/103600 (PMC13592749; doi:10.2196/103600)
Supplement: Supplementary file 1 [file rehab-v13-e103600-s002.docx]

| **Table 1. Characteristics of Commercially Insured and Medicare Advantage Patients Using Remote Therapeutic Monitoring (RTM)** | | | | | | |
| --- | --- | --- | --- | --- | --- | --- |
|  | **Patients Using RTM**  **2022-2024**  **(n=16,116)** | | **Patients Using RTM**  **during 1 Year**  **(n=15,229)** | | **Patients Using RTM**  **during 2 or More Years**  **(n=887)** | |
|  | **Commercial Insurance**  **(n=12,951)** | **Medicare Advantage**  **(n=3,165)** | **Commercial Insurance**  **(n=12,280)** | **Medicare Advantage**  **(n=2,949)** | **Commercial Insurance**  **(n=671)** | **Medicare Advantage**  **(n=216)** |
| **Age** | | | | | |  |
| 0-18, n (%) | 642  (5%) | 49  (1.5%) | 592  (4.8%) | 41  (1.4%) | 50  (7.5%) | 8  (3.7%) |
| 19-34, n (%) | 1,895  (14.6%) |  | 1822  (14.8%) |  | 73  (10.9%) |  |
| 35-44, n (%) | 2,775  (21.4%) |  | 2634  (21.4%) |  | 141  (21%) |  |
| 45-54, n (%) | 3,523  (27.2%) |  | 3317  (27%) |  | 206  (30.7%) |  |
| 55-64, n (%) | 4,116  (31.8%) |  | 3915  (31.9%) |  | 201  (30%) |  |
| 65-74, n (%) | - | 1,462  (46.2%) | - | 1,342  (45.5%) | - | 120  (55.6%) |
| 75+, n (%) | - | 1,654  (52.3%) | - | 1,566  (53.1%) | - | 88  (40.7%) |
| **Sex** | | | | | |  |
| Female, n (%) | 8,061  (62.2%) | 1,982  (62.6%) | 7,663  (62.4%) | 1,844  (62.5%) | 398  (59.3%) | 138  (63.9%) |
| Male, n (%) | 4,890  (37.8%) | 1,183  (37.4%) | 4,617  (37.6%) | 1,105  (37.5%) | 273  (40.7%) | 78  (36.1%) |
| **Region** | | | | | |  |
| South, n (%) | 6,112  (47.2%) | 522  (16.5%) | 5,843  (47.6%) | 472  (16%) | 269  (40.1%) | 50  (23.1%) |
| West, n (%) | 3,272  (25.3%) | 247  (7.8%) | 3,003  (24.5%) | 225  (7.6%) | 269  (40.1%) | 22  (10.2%) |
| Northeast, n (%) | 2,003  (15.5%) | 613  (19.4%) | 1,911  (15.6%) | 567  (19.2%) | 92  (13.7%) | 46  (21.3%) |
| North Central, n (%) | 1,554  (12%) | 1,775  (56.1%) | 1,514  (12.3%) | 1,677  (56.9%) | 40  (6%) | 98  (45.4%) |
| **Insurance** | | | | | |  |
| PPO, n (%) | 7,855  (61%) | 2,619  (82.8%) | 7,473  (61.2%) | 2,444  (82.9%) | 382  (57.6%) | 175  (81%) |
| HDHP, n (%) | 1,903  (14.8%) | - | 1,820  (14.9%) | - | 83  (12.5%) | - |
| HMO, n (%) | 826  (6.4%) | 379  (12%) | 775  (6.3%) | 346  (11.7%) | 51  (7.7%) | 33  (15.3%) |
| Other, n (%) | 2,293  (17.8%) | 1  (<1%) | 2,146  (17.5%) | 0  (0%) | 147  (22.2%) | 1  (0.5%) |
| **CCI Score** | | | | | |  |
| 0-1, n (%) | 12,801  (98.8%) | 2,768  (87.5%) | 12,133  (98.8%) | 2,570  (87.1%) | 668  (99.6%) | 198  (91.7%) |
| 2-3, n (%) | 140  (1.1%) | 360  (11.4%) | 137  (1.1%) | 342  (11.6%) | 3  (<1%) | 18  (8.3%) |
| 4+, n (%) | 9  (<1%) | 37  (1.1%) | 9  (<1%) | 37  (1.3%) | 0  (0%) | 0  (0%) |
| ^a^ - = Not applicable  ^b^ Other: Comprehensive, Point of Service, Consumer-Directed Health Plan, Exclusive Provider Organization  ^c^ PPO: Preferred Provider Organization, HDHP: High-Deductible Health Plan, HMO: Health Maintenance Organization  ^d^ CCI 0-1 is categorized as low risk, 2-3 as moderate risk, and 4+ as high risk  Unknown and missing data were excluded from the table. Region data were unknown for 10 (<1%) commercially insured and 8 (<1%) Medicare Advantage patients in the overall cohort. Insurance types were missing for 166 Medicare Advantage patients (5.2%) in the overall cohort | | | | | | |

| **Table 2. Clinician Specialties and Facilities** | | |
| --- | --- | --- |
| **Provider Category** | **Provider Type** | **STD PROV** |
| Primary Care | Family Practice | 240 |
|  | Internal Medicine | 204 |
|  | Pediatrician (NEC) | 400 |
| Medical Specialties | Cardiovascular Disease/Cardiology | 250 |
|  | Hematology | 280 |
|  | Oncology | 380 |
|  | Nephrology | 290 |
|  | Rheumatology | 300 |
|  | Endocrinology & Metabolism | 270 |
|  | Pulmonary Disease | 295 |
|  | Geriatric Medicine | 245 |
|  | Gastroenterology | 275 |
|  | Critical Care Medicine | 265 |
|  | Pediatric Critical Care Medicine | 423 |
|  | Allergy & Immunology | 230 |
|  | Palliative Medicine | 227 |
|  | Pediatric Specialist (NEC) | 410 |
| Surgeons | Surgeon (NEC) | 500 |
|  | Colon & Rectal Surgery | 510 |
|  | Neurological Surgery | 520 |
|  | Orthopaedic Surgery | 530 |
|  | Otolaryngology | 340 |
|  | Obstetrics & Gynecology | 320 |
|  | Ophthalmology | 330 |
|  | Pediatric Ophthalmology | 415 |
|  | Pediatric Orthopaedics | 418 |
|  | Urology | 210 |
|  | Sports Medicine (Pediatrics) | 453 |
| Other Physicians | Preventative Medicine | 360 |
|  | Neurology | 260 |
|  | Pain Medicine | 140 |
|  | Psychiatry | 365 |
|  | Anesthesiology | 150 |
|  | Radiology | 180 |
|  | Podiatry | 130 |
|  | Emergency Medicine | 220 |
|  | Multi-Specialty Physician Group | 206 |
|  | Medical Doctor (MD) | 200 |
|  | Child Psychiatry | 458 |
|  | Physical Medicine & Rehab | 350 |
|  | Osteopathic Medicine | 202 |
| APP | Physician Assistant | 845 |
|  | Nurse Practitioner | 825 |
| Other Non-Physicians | Optometrist | 830 |
|  | Medical Technician | 815 |
|  | Nursing Services | 822 |
|  | Psychiatric Nurse | 824 |
|  | Nurse Anesthetist | 827 |
|  | Case Manager | 960 |
|  | Therapy (Physical) | 850 |
|  | Dietitian | 810 |
|  | Chiropractor/DCM | 120 |
|  | Psychologist | 860 |
|  | Acupuncturist (Alternative/Holistic) | 865 |
|  | Therapists (Supportive) | 853 |
|  | Therapists (Alternative) | 855 |
|  | Health Educator/Agency | 900 |
| Facility Based | Acute Care Hospital | 1 |
|  | Rehabilitation Facility | 25 |
|  | Mental Health/Chemical Dependency Facility | 20 |
|  | Special Care Facility (NEC) | 42 |
|  | Other Facility (NEC) | 40 |
|  | Home Health Organization/Agency | 920 |
|  | Public Health Agency | 950 |
|  | Supply Center | 940 |
|  | Laboratory | 930 |
|  | Hearing Labs | 915 |
|  | Mental Health/Chemical Dependency Facility (NEC) | 23 |
